# Medical Schools in Ireland

**Published:** 1921-08-27

**Authors:** 


### MEDICAL SCHOOLS IN IRELAND.

**University of Dublin (Trinity College).**—The Medical School of Trinity College, or School of Physic in Ireland, was established in 1711. All classes are open to external students as well as to students of the University, provided that they have passed an examination in Arts recognised by the General Medical Council. Women students are admitted to the classes and to the University degrees. Candidates for the degrees in Medicine (M.B.), Surgery (B.Ch.), and Midwifery (B.A.O.) must be of B.A. standing, and must have spent at least five academic years, reckoned from the date of Matriculation, in the study of Medicine. The Arts course may be taken concurrently with the Medical course, and the B.A. degree need not be taken before the final Medical examination, but the Medical degrees are not conferred without the Arts degree. The M.D. degree is awarded to graduates in Medicine of M.A. standing who have read and discussed an approved thesis publicly before the Regius Professor of Physic and his assessor. Special classes are held for the Diploma in Public Health. The winter session begins on October 3. Further information regarding the Medical and Dental courses may be obtained by application to the Registrar, School of Physic, Trinity College, Dublin.

**Royal College of Surgeons in Ireland (the Schools of Surgery).**—These schools are carried on within the college buildings, and are specially subject to the supervision and control of the Council. The buildings have been reconstructed. There are special rooms set apart for lady students. The Medical Students' Guide will be forwarded post free on application to the Registrar, Royal College of Surgeons, Stephen's Green, West Dublin.

**University College Medical School, Dublin.** Founded in 1855, it is now the largest medical school in Ireland. It prepares students specially for the examinations of the National University and the Conjoint Colleges of Ireland and Edinburgh, but its lectures and practical courses are recognised by all the licensing bodies in Great Britain and Ireland. In addition to the ordinary Medical examinations it prepares students for the D.P.H. and for the various higher University examinations in pathology, physiology, chemistry, etc. Eight exhibitions and numerous gold and silver medals are offered annually for competition.

### BELFAST.

**Queen's University.**—This University provides all the classes required for a complete medical curriculum. Women are eligible as students on exactly the same terms as men. Clinical instruction is given at the Royal Victoria Hospital, which was rebuilt a few years ago and has 300 beds, and at the Mater Infirmorum Hospital, which has 150 beds. Various other hospitals are open to the students of the University. The cost of the curriculum intended for students proceeding to the degrees of the Queen's University of Belfast is approximately £130. This includes examination fees and a perpetual ticket for attendance at the Royal Victoria Hospital or the Mater Infirmorum Hospital, and fees for the special hospitals. The course for the Conjoint Board costs about the same amount. The matriculation regulations and a pamphlet containing full information regarding entrance and other scholarships, etc., can be had free of cost on application to the Secretary, Queen's University, Belfast. The calendar is published in September; price 2s. A Department of Dentistry was opened in 1920-21, in which students may study for L.D.S., B.D.S., and M.D.S.

### CORK.

**University College.**—Students can attend courses which meet the requirements of the National University of Ireland, the Conjoint Boards of London, Edinburgh, or Dublin, and other examining bodies. Clinical instruction is given at the North and South Infirmaries, each of which contains 100 beds, at the District Hospital, which contains 1,200 beds, and at the Mercy Hospital, which contains 130 beds. There are also available for students the Eye, Ear, and Throat Hospital (35 beds), Cork Fever Hospital, Erinville Lying-in Hospital, Cork Maternity Hospital, Victoria Hospital, etc. The college is a constituent college of the National University of Ireland, and holds its own examinations in all subjects required for the degrees of that University. At least thirty-six Entrance and County Council Scholarships, varying from £50 to £20, which may be held in any Faculty, are offered for competition. There are, in addition, twelve scholarships of about £30 each offered for competition to medical students of the second, third, fourth, and fifth years. The Blayney Scholarship, of about £32, is awarded to the candidate who, being of the prescribed standing, obtains highest marks with honours at the National University examination for the M.B., B.Ch., B.A.O. degrees, taken as a whole. There is in the College a special course for medical graduates wishing to qualify for the D.P.H. There is also a fully equipped Dental Hospital, where students are prepared for the degrees in Dental Surgery of the University. Further information can be had on application to the Registrar, University College, Cork.

### GALWAY.

**University College.**—There are nine scholarships of the annual value of £25 each, specially reserved for students in medicine, and awarded on the results of the University examination of the corresponding year. In addition, medical students at entrance may compete at the General Entrance Scholarship examination, and hold the scholarship, if successful, in the Faculty of Medicine. Clinical instruction is given in the Galway County Hospital and in the Galway Union Tuberculosis and Fever Hospitals. Full information as to courses of lectures, scholarships, and class fees can be obtained from the Registrar, University College, Galway.

